# Reviews and Book Notices

**Published:** 1878-09

**Authors:** 


### Reviews and Book Notices.

---

ETUDE EXPERIMENTALE ET COMPARÉE SUR L'ARSENIC ET L'HUILE DE FOIE DE MORUE DANS LE TRAITEMENT DE LA PHTHISIE PULMONAIRE. Par Joanny Rendu, Paris, G. Masson, 1878.

EXPERIMENTAL AND COMPARATIVE STUDY OF ARSENIC AND COD LIVER OIL IN PHTHISIS PULMONALIS. By Joanny Rendu.

This is a pamphlet of 48 octavo pages of text and ten plates, eight of which represent the curves of temperature, sweat, diarrhœa, muscular force and weights of the patients, and two the thermometric tracings. The study was made in the *Hôtel-dieu* at Lyons, in 1877, the special object being to determine the relative values of arsenic and cod liver oil in the second and third stages of phthisis. The following are the author's conclusions:

I. *Practical Conclusions.*—To determine which of the two remedies is preferable in the treatment of phthisis, he took thirty-five patients in the 2d and 3d stages, and placed them at hazard in three classes: 1st, those placed under simple tonic treatment, otherwise called the expectant treatment; 2d, those treated with arsenic, and 3d, those with cod-liver oil. These patients were, for the most part, followed for two and a half or three months.

Every ten days their *exact* weight was taken, and the amount of their *muscular force* by the dynamometer. Note was also made of the degree of *sweating* and *diarrhœa*, and each day, morning and evening, the *rectal temperature* was taken. For various reasons it became necessary during the observation, to omit ten of these cases to avoid possible error. The remaining twenty-five cases were divided into three classes, eight in the first, eleven in the second, and six in the third.

Of the eight patients subjected to the expectant treatment, six lost in weight and two gained; of the eleven treated by arsenic, ten lost in weight and only one gained; of the six treated by cod liver oil, two lost in weight and four gained.

The degree of gain or loss by these three methods of treatment may be appreciated in the following manner, the duration of the experiments and the weight of the patients being supposed the same for the three classes, we find:

By the expectant plan a loss of 4 k. 965 g. for 100 k. of patient.

By the arsenic treatment a loss of 4 k. 703 g. for 100 k. of patient.

By cod liver oil treatment, a *gain* of 2 k. 150 g. for 100 k. of patient.

Or rather by adding to this last series, as it is legitimate to do, *what has not been lost*, to what has been gained, we find by the use of cod liver oil a *real increase of more than* 7 *kilograms for* 100 *k. of the patient.* Arsenic gave only the results of the simple expectant plan. On the contrary, the patients who took cod liver oil, alone gained in weight and strength. The pulmonary lesion did not appear to be as happily influenced by the cod live oil, as the general state and the nutrition of the patient. The medicine has little or no effect when the pulmonary lesion is considerable.

II. *Scientific Conclusions.*—The comparative study of the curves of sweating, diarrhœa, temperature, weight and muscular force of the patients, leads to the following results:

There is constantly an inverse relation between the weight and the temperature.

The same inverse relation exists between the weight on the one side and the sweating and diarrhœa on the other side.

The weight and muscular force are in direct relation. When here is neither fever, sweating nor diarrhœa, the weight increases progressively in relation with the treatment followed, but this increase is much more rapid if the patient be not a consumptive.

If the medium temperature remains in the neighborhood of 38° without being accompanied by sweating or diarrhœa, the patient may still keep up and remain so for some time; in any case he loses but little.

The increase of weight begins about fifteen days after the temperature has come down to the normal level.

There exists an inverse relation between the temperature, on one side, and the sweating and diarrhœa on the other; one may have these complications combined two by two, but they are not seen all three at once. L. W. C.

A PRACTICAL TREATISE ON AURAL SURGERY. By H. Macnaughton Jones, M. D., M. Ch., F. R. C. S., I. and Edin. Philadelphia: Lindsay & Blakiston. 1878. Chicago: Jansen, McClurg & Co.

Every now and then small books are forced upon the medical profession under the alluring title of "Practical Treatises," which pretend to furnish, in the most condensed form, a complete résumé of our present knowledge in this or that branch of medical science. They are a sort of condensed milk; yet unlike the latter they cannot be made palatable and digestible by dilution, but must be taken in the crude and indigestible state in which they are presented.

The "Practical Treatise" before us is no exception to the rule, nor does it give us a high opinion of the author's literary talent. It is composed without system and written with haste, leaving some of the most modest demands unsatisfied.

The author's apology for publishing this book is that which we find in all these practical treatises, "intended chiefly for the busy practitioner and student," and from this stand-point we shall examine his fabrication.

We believe that in a small compendium, wood-cuts should be reduced to the smallest possible number of absolutely necessary illustrations. But to ornament 172 small octavo pages with forty-five pictures of such instruments as nasal specula, syphon douche, nasal douche, syringes, etc., is a pictorial extravagance not in keeping with the modest space left for the text.

One hundred pages are devoted to the modes of examining the ear and the description of instruments. In this part the author's remarks are clear and to the point; and only his hasty writing could make him commit this gross blunder, on page 33, of letting the triangular light spot of the membrana tympani begin at the *short* process, instead of at the extremity of the long process or handle of the hammer.

Only seventy-two pages are reserved for the consideration of the maladies of the ear. The eminently *practical* use the author of this "practical" treatise, undertook to make of this limited space, is manifested by the fact that ten pages are devoted to the description of "Othæmatoma," while only two pages are allowed for "Polypi!" And the commonest and practically most important aural diseases, the catarrhal affections of the middle ear and Eustachian tube, are disposed of on *five pages* (*!*) when nine pages could be given up for a quotation from a paper on "Deaf-mutism" which was read by Dr. Turnbull before the International Congress.

Another curious illustration of the author's peculiar idea respecting a "practical" book, may here be mentioned. On page 79 we find these remarks: "When and how are we to incise the membrane and make an artificial opening into the tympanum? This is just one of those disputed points which I determined to avoid dwelling on in this work, intending it as I do for practitioners whose minds should not, if possible, be burthened about operations which they are not likely to be called on to perform, and the decision as to the propriety of which involves a more lengthened and varied experience than is likely to fall to the lot of most surgeons." A few pages further on, the author has already forgotten this determination to avoid burdening the minds of practitioners "about operations which they are not likely to perform," etc., etc., for he devotes *thirteen pages* to "Tenotomy of the tensor tympani," although he looks upon it as a questionable operation, and acknowledges his total ignorance about it with more candor than grammatical finish: "I wish to remark that this is an operation the utility of which I have myself no practical experience. I have hesitated to practice tenotomy until more definite results are obtained from its performance, and the cases in which it is indicated are more clearly defined."

In view of the fact that America has produced three excellent text books on aural surgery by Roosa, Turnbull and Burnett, it is difficult to understand how a leading American publisher could be found who would reproduce an inferior work like the one before us; for there is no valid reason for its existence. If a practitioner is too busy, or a student too lazy, to read one of our good and complete text books on Otology, he should never meddle with aural diseases, nor consent to practice on such cases with the diffuse knowledge to be gained from the reading of these small, "dime novel" volumes styled "Practical Treatises." F. C. H.

THE ATLANTIC ISLANDS AS RESORTS OF HEALTH AND PLEASURE. By S. G. W. Benjamin, author of "Contemporary Art in Europe," etc. Illustrated. New York: Harper Bros., 1878. Chicago: Jansen, McClurg & Co.

This book is a very attractive narration of the author's recent visit to the following islands of the North Atlantic: The Bahamas, the Azores, the Channel Islands, the Magdalen Islands, Maderia, Teneriffe, Newfoundland, the Bermudas, Belle Isle-en-mer, Prince Edward's Island, Isles of Shoals, Cape Breton Island the Isle of Wight.

An appendix furnishes, in the language and style of a guide-book, copious information regarding the attractions of each island for both invalids and sportsmen, sanitary statistics, means for reaching these resorts, and the hotels and expenses of living.

The book is very liberally illustrated and tastefully finished, and will be an entertaining lecture to every one. In those who have visited the Atlantic isles it will revive pleasant reminiscences; and those who have never been there, will receive such vivid impressions that they will be inclined to believe they have made the journey in reality. F. C. H.

PERSONAL APPEARANCE AND THE CULTURE OF BEAUTY, WITH HINTS AS TO CHARACTER. By T. S. Sozinskey, M. D. Ph. D. Philadelphia; pp. 196. Price, $1.50.

The book is one which commends itself as a work on hygiene, offering as a reward for the fulfillment of each and every law, the attainment, so far as nature's outlines will permit, of personal beauty, the outlines even being often amenable to improvement and correction, as measured by the standard of beauty. The author gives the standard type of beauty, male and female, and gives the geometrical rule of procedure (Fechner, Dürer), on which nature operates in producing symmetry of form, as illustrated in art by Apollo and Venus.

Beauty being an improvable heritage, the author gives various suggestions relative to the cultivation of the various features, the forehead, eyebrows, eyes, nose, cheeks, chin, lips, teeth, lines, wrinkles, complexion and hair, and concludes with some sensible remarks on dress. A novel instrument is introduced whose use is to modify the growing infant's nose, called a nose machine. By it the unduly low-bridged nose is lifted up; and various tilts are corrected. There will exist a prejudice on the part of mothers to this invention, as it destroys much of the poetry in the sleeping cherub faces, to see clothes-pins attached to the little noses. But such prejudices should be subdued inasmuch as, according to Lavater, "A nose physiologically good is of unspeakable weight in the balance of physiognomy." Some observations are given on the import of features, enabling the observer to gain some glimpse of character, none of which are new. The book is fairly worth reading. S. F. B.

Urological Dictionary, containing an explanation of numerous technical terms; the qualitative and quantitative methods in urinary investigations; the chemical characters and microscopic appearances of the normal and abnormal elements of the urine and their clinical indications. By John King, M. D. With twenty-seven useful tables and thirty-nine wood-cuts. Cincinnati: Wilstach, Baldwin & Co. Chicago: Jansen, McClurg & Co.

The above book lays before us, in alphabetical order, almost all that is of any importance which has been written relative to the physiological and pathological peculiarities of the urine. Much credit is due to Dr. King for getting together the very latest methods devised to render urinalysis practical and available to the busy practitioner. We observe among other devices he has given due force to the specific gravity method of determining the quantity of urea, devised by Dr. Geo. B. Fowler, of New York; and has inserted in the supplement the very simple method of determining approximately the quantity of albumen, suggested by Dr. Wm. Roberts, of Manchester, England. It is certainly the most complete treatise on urinalysis extant, and we are convinced that those who examine judiciously the various works published, in order to make a selection will not hesitate long in making their choice.

There is, however, one thing which disappointed us. The derivation of words is entirely ignored. In a dictionary so well compiled we think we had a right to expect the derivation of such words as pycnometer, pyoturia and the like. Although the advisability of using classical words to express modern ideas may be called in question, yet if we use them we want to know as much as possible about them, so as to associate them intimately with their respective ideas.

The appendix also contains valuable information in useful tabular forms. It consists of 266 pages, and the binding is strong and durable, such as a dictionary requires. R. T.

Transactions of the Medical Association of Georgia. Twenty-ninth Annual Session. Atlanta, April 17, 18, 19, 1878.

This volume presents, in a nicely finished, thoroughly revised form, the scientific labors of the Georgia State Medical Society, and contains some highly interesting essays. Dr. Le Hardy, at great length, discusses the history, causes, nature, pathology and treatment of yellow fever, from his personal experience during the epidemic of 1876, in Savannah. His observations fortified his belief in the *local* origin of yellow fever. His propositions are: 1. That yellow fever can *originate* here under certain conditions of earth and air. 2. That the sanitary condition of the low lands surrounding the city *is the most important factor* in the production of our yellow fever. 3. That *importation* is not necessary for its production, and, therefore, the strictest quarantine of ships, vessels or railroad trains will not prevent its occurrence in this place. Quarantine should be abolished and for it should be substituted *modern scientific disinfection*, which, in a few hours, will remove all causes of infection from any ship.

Dr. A. W. Calhoun, Atlanta, gives a report of one hundred and thirty operations for strabismus. One hundred and sixteen operations were for convergent squint, and only fourteen involved the external rectus muscle, constituting divergent squint. As to the sex, the patients were pretty equally divided, viz., sixty-eight females and sixty-two males. But it is a singular fact, that among the whole number of cases only three have occurred amongst the negroes, although they have been collected in the midst of the densest portion of the black population of the South.

*A new uterine tent* is offered by Dr. W. T. Goldsmith, Atlanta. It is the pith of the dried cornstalk. "You take a joint of the dried stalk, strip it of its cuticle, and compress the pith, slowly and firmly, between the thumb and index finger. * * * By continued pressure you easily reduce it to four or five times less its original size. You can compress it to any intermediate size; or it may be used without compression, to carry medicaments to the interior of the uterus. Because of its ready compression to any desired bulk, you have the tent entirely under your control. Slight compression will give you moderate dilating power. Compress it as much as possible and you can get a dilating power equal to the sea-tangle or sponge. You may compress the pith first, and afterward, with a sharp knife, trim to the desired length and size; or you may first cut the pith to the size you wish it to dilate, and then compress it for easy introduction. * * The pith absorbs fluids readily, and when compressed, expands by such absorption to its original size." A few typical cases are reported, to illustrate the use and advantages of this new tent.

Dr. William A. Love briefly alludes to the diagnostic value of the appearance of the hard and soft palate in intestinal diseases. "In all that class of diseases in which the general condition of the system demands the use of remedies known as *cholagogues* * * * experience has taught me that I risk nothing in saying that the muco-periosteal membrane in the roof of the mouth, will, by its yellow tinge, invariably indicate the necessity for their administration. *Per contra*, I may say, with equal confidence, that the absence of this yellowness indicates, with equal certainty, that such a class of remedies has been sufficiently used, or is not needed." The writer also alludes to the fact that in the exanthematous fevers, the eruption appears in the roof of the mouth from twelve to twenty-four hours earlier than on the cutaneous surface.

F. C. H.

Fownes' Manual of Chemistry, Theoretical and Practical. Revised and corrected by Henry Watts, B. A., F. R. S., editor of the *Journal of the Chemical Society;* author of a Dictionary of Chemistry, etc. A new American edition, from the twelfth English edition. Edited by Robert Bridges, M. D., Professor of Chemistry in the Philadelphia College of Pharmacy. With one hundred and seventy-seven illustrations. Cloth, pp. 1027. Philadelphia: H. C. Lea. Chicago: Jansen, McClurg & Co.

This standard classical work on chemistry needs no recommendation to those who are acquainted with the literature of chemistry. It has, as the twelfth edition will testify, for years been a text book of great value, and the energy displayed by the editors in keeping it abreast of the times will enable it to retain the position in chemical literature which its merits have secured.

The changes introduced are mostly in the organic department, special attention being paid to the study of Isomerism, making the addition quite important.

